# Towards pharmacogenomics-guided tuberculosis (TB) therapy: N-acetyltransferase-2 genotypes among TB-infected Kenyans of mixed ethnicity

**DOI:** 10.1186/s12920-023-01788-1

**Published:** 2024-01-06

**Authors:** Lilian N. Njagi, Jared O. Mecha, Marianne W Mureithi, Leon E. Otieno, Videlis Nduba

**Affiliations:** 1 Centre for Respiratory Disease Research, Kenya Medical Research Institute, Nairobi, Kenya; 2 Department of Medical Microbiology & Immunology, Faculty of Health Sciences, University of Nairobi, Nairobi, Kenya; 3 Department of Clinical Medicine and Therapeutics, Faculty of Health Sciences, University of Nairobi, Nairobi, Kenya; 4 Molecular Medicine and Infectious Disease Laboratory, University of Nairobi, Nairobi, Kenya

**Keywords:** Acetylation, *N*-acetyltransferase-2 (NAT2), Genetic variants, Tuberculosis, Pharmacogenetics

## Abstract

**Background:**

Though persons of African descent have one of the widest genetic variability, genetic polymorphisms of drug-metabolising enzymes such as N-Acetyltransferase-2 (*NAT2*) are understudied. This study aimed to identify prevalent *NAT2* single nucleotide polymorphisms (SNPs) and infer their potential effects on enzyme function among Kenyan volunteers with tuberculosis (TB) infection. Genotypic distribution at each SNP and non-random association of alleles were evaluated by testing for Hardy-Weinberg Equilibrium (HWE) and Linkage Disequilibrium (LD).

**Methods:**

We isolated genomic DNA from cryopreserved Peripheral Blood Mononuclear Cells of 79 volunteers. We amplified the protein-coding region of the *NAT2* gene by polymerase chain reaction (PCR) and sequenced PCR products using the Sanger sequencing method. Sequencing reads were mapped and aligned to the NAT2 reference using the Geneious software (Auckland, New Zealand). Statistical analyses were performed using RStudio version 4.3.2 (2023.09.1 + 494).

**Results:**

The most frequent haplotype was the wild type NAT2^*^4 (37%). Five genetic variants: 282C > T (NAT2^*^13), 341 T > C (NAT2^*^5), 803A > G (NAT2*12), 590G > A (NAT2*6) and 481C > T (NAT2*11) were observed with allele frequencies of 29%, 18%, 6%, 6%, and 4% respectively. According to the bimodal distribution of acetylation activity, the predicted phenotype was 76% rapid (mainly consisting of the wildtype NAT2^*^4 and the NAT2*13A variant). A higher proportion of rapid acetylators were female, 72% vs 28% male (*p* = 0.022, odds ratio [OR] 3.48, 95% confidence interval [CI] 1.21 to 10.48). All variants were in HWE. *NAT2* 341 T > C was in strong complete LD with the 590G > A variant (D′ = 1.0, r^2^ = − 0.39) but not complete LD with the 282C > T variant (D′ = 0.94, r^2^ = − 0.54).

**Conclusion:**

The rapid acetylation haplotypes predominated. Despite the LD observed, none of the SNPs could be termed tag SNP. This study adds to the genetic characterisation data of African populations at *NAT2,* which may be useful for developing relevant pharmacogenomic tools for TB therapy. To support optimised, pharmacogenomics-guided TB therapy, we recommend genotype-phenotype studies, including studies designed to explore gender-associated differences.

## Background

Polymorphisms in genes encoding for drug metabolising enzymes can alter enzymatic activity and, as a result, alter therapeutic effectiveness and toxicity. The N-Acetyltransferase-2 (*NAT2*), a phase II xenobiotic enzyme, is responsible for the biotransformation of many aromatic and heterocyclic amines, such as Isoniazid (INH). The gene encoding the enzyme is located on chromosome 8 at p22. Genetic variability in N-acetylation capacity is a well-known phenomenon attributable to the polymorphisms in the *NAT2* gene in humans [1, 2]. There are over 65 allelic variants to the reference NAT2*4, each possessing one or more single nucleotide polymorphisms (SNPs) in the *NAT2* loci. The most frequent SNPs (presented by the reference SNP cluster identifiers (rsID) and nucleotide change) are rs1799929 (481C > T), rs1799930 (590G > A), rs1799931 (857G > A), rs1801279 (191G > A), rs1041983 (282C > T), rs1801280 (341 T > C) and rs1208 (803A > G), with varying proportions in different populations [3–7]. These allelic variants, determined from genotypic analyses, confer rapid/fast (2 rapid alleles) and slow (two slow alleles) enzymatic activity/phenotypic effects.

Genotypic analyses have implications for precision medicine, whereby pharmacogenomics-guided therapy has been shown to result in improved clinical effectiveness [8, 9]. Tuberculosis (TB) remains an infectious disease of global public health significance decades after the discovery of effective chemotherapy. INH, a core drug in treating TB, is subject to a bimodal distribution of metabolism, fast and slow, resulting from the variability in the *NAT2* gene. People with fast enzymatic activity are prone to suboptimal drug levels, resulting in reduced efficacy, [10] and a possible contribution to the evolution of drug-resistant TB [9]. People with slow enzymatic activity are at an increased risk of drug toxicity [9]. This alteration of treatment effectiveness underscores the need for pharmacogenomics-guided TB therapy [1, 9–11].

Understanding population genetics is essential for pharmacogenomics-guided TB therapy, given that the *NAT2* gene is a target for population-specific selection pressure. Extensive studies have demonstrated significant population-specific polymorphisms in the *NAT2* gene. However, many of these were conducted among Asians and Caucasians [8, 12–16]. Extrapolating these data to the African population results in misrepresentation. Although pharmacogenomic studies among Africans are sparse, interethnic genetic diversity is prevalent [6, 7, 11, 12, 17–23]. This paper presents the results of a study that sequenced the *NAT2* gene to identify prevalent SNPs and inferred their potential effects on enzyme function among people with TB infection in Kenya. The heterogeneity of this population and the possibility of recombination among the SNPs were also evaluated.

## Materials and methods

### Study setting and design

This cross-sectional study used data collected at enrolment into a prospective observational study of adults aged ≥18 years from three HIV care and prevention centres in Nairobi, Kenya. The clientele attending all three facilities is cosmopolitan. Laboratory procedures occurred at the Kenya Medical Research Institute, Center for Respiratory Disease Research (KEMRI CRDR) immunology laboratory and the Molecular Medicine and Infectious Diseases Laboratory of the University of Nairobi, Kenya.

### Study period and population

Participants seeking HIV care and prevention services between December 2019 and December 2020 were eligible to enrol in the study. They were all of African descent from Kenya. A total of 79 participants with TB infection confirmed by a positive interferon-gamma release assay consented to genetic testing. The sample size was calculated to estimate the frequency of the least common alleles in the population reliably. Thus, it was determined based on a 0.4% allele frequency representing NAT2*7, [21, 24] one of the major known *NAT2* genetic variants, [25] and one of the least common alleles in many African ethnic groups, [21, 24, 26] at a 95% confidence level.

### Sample collection, preparation and DNA extraction

Blood samples for genotyping were obtained from 79 participants. Peripheral mononuclear cell samples (PBMCs) were extracted, cryopreserved at − 80 °C, and batched for genetic analysis. DNA extraction was done using the Qiagen DNA Mini Kit (Hilden, Germany) per the manufacturer’s instructions. Briefly, after thawing, 200ul of PBMCs were lysed using 200ul Lysis Buffer AL and 20ul of Proteinase K and incubated at 56 degrees Celsius for 10 minutes. For DNA precipitation, 200ul of 100% molecular-grade ethanol was added to the lysate. The lysate was then transferred to a sterile affinity spin column and spun at 6800 g for nucleic acid target capture, then washed with Wash Buffer A1 and A2, respectively, centrifuged at 6800 g and 17,000 g for 1 min, and eluted in 40ul of sterile RNAase-free water. DNA was stored at − 20 degrees for subsequent use.

### N-acetyltransferase 2 gene amplification

The coding region (exon2) of the *NAT2* gene was amplified by the Polymerase Chain Reaction (PCR) technique using the 96-Well Fast Thermal Cycler (Applied Biosystems, Waltham, Massachusetts, United States). Gene-specific primer set (Inqaba Biotec South Africa), forward 5’GGGATCATGGACATTGAAGCA3’ and reverse 5’ATGTTTTCTAGCATGAATCACTCTG3’ as described previously [27] were used, amplifying a total fragment length of about 1150 bp. The final PCR reaction volume (25ul) consisted of 3ul of genomic DNA, 2X Mastermix (2X Green GoTaq™ Reaction Buffer pH 8.5, 400 PM dATP, 400 PM dGTP, 400 PM dCTP, 400 PM dTTP and 3 mM MgCl2), and 10 picomoles of forward and reverse primers. The PCR conditions used are as outlined by Zahra et al. [27] Initial denaturation was set at 95 °C for 3 minutes, followed by 35 cycles consisting of a denaturation step at 95 °C for 30 seconds, an annealing step at 60 °C for 30 seconds, and an elongation step at 72 °C for 1 minute. This was completed with a final elongation cycle at 72 °C for 5 minutes [27].

### Agarose gel electrophoresis and purification

To verify amplification of the *NAT2* coding region, the PCR products were run on a 1% agarose gel stained with SYBR™ safe DNA gel stain (Thermo Fisher Scientific, San Francisco, USA) and visualised using UVTEC™ Gel Documentation System (Cleaver Scientific, United Kingdom). The amplified PCR fragment was purified using EXOSAP-IT™ (Applied Biosystems, Waltham, Massachusetts), whose function is the hydrolysis of excess primers and nucleotides. Briefly, PCR tubes were labelled appropriately, and 2 μl of EXOSAP-IT™ was added to each tube. To the corresponding tube, 5 μl of the initial PCR product was slowly added and incubated at 37 °C for 15 minutes following enzyme inactivation, which occurred at 80 °C for 15 minutes.

### N-acetyltransferase 2 gene sequencing

The exon 2 coding region of the *NAT2* gene was sequenced using Brilliant-Dye Terminator v3.1 Cycle Sequencing kit (Nimagen, Netherlands). Three microlitres (3ul) of purified PCR product was added to 7.0ul sequencing mix comprising 5X Sequencing Buffer, Brilliant Dye Terminator, 10 picomoles of forward primer 5’GGGATCATGGACATTGAAGCA3’ and a separate reaction consisting of a reverse primer 5’ATGTTTTCTAGCATGAATCACTCTG 3′ [27]. The cycling sequencing conditions were 25 cycles of 96 °C for 10 seconds, 50 °C for 5 seconds, and 60 °C for 4 minutes. Cycle Sequencing products were purified using BigDye-Xterminator Purification Kit (Applied Biosystems, Waltham, Massachusetts). Using Applied Biosystem’s 3730XLGenetic Analyzer, Sanger sequencing was performed, and AB1 files containing chromatogram data were generated.

### Sequence data analyses

To screen for different SNPs, alignment of forward and reverse sequences was done separately and mapped to the *Homo sapiens* N-acetyltransferase 2 (*NAT2*) reference genome GenBank accession number NG012246 [28]. This is the NAT2*4 reference historically designated as wild type. Sequences of samples were evaluated for chromatogram peaks and overlapping bases to make consensus sequences. To ensure the accuracy of identifying homozygous and heterozygous SNPs, independent validation and interpretation of chromatogram data was conducted using bioinformatics data analysis software (Geneious Prime Dotmatics, Auckland, New Zealand). The homozygous and heterozygous SNPs were differentiated depending on electropherogram peaks and quality scores. Single clear peaks represented a homozygous SNP, whereas double peaks with different fluorescent peaks indicated a heterozygous SNP.

### Acetylator phenotype classification

*NAT2* enzymatic activity populations were classified into two acetylation phenotypes, slow and rapid, according to the Human *NAT2* Alleles (Haplotypes) nomenclature [29]. The alleles considered rapid were the wild-type *NAT2∗4* and SNPs of 282C > T (*NAT2∗13*), 481C > T (*NAT2∗11*), and 803A > G (*NAT2∗12*) [29, 30]. All other alleles containing SNPs of 341 T > C (*NAT2∗5*), 590G > A (*NAT2∗6*), 857G > A (*NAT2∗7*) and 191G > A (*NAT2∗14*) were considered slow [29–31].

### Statistical analysis

Statistical analysis was performed using RStudio: Integrated Development Environment for R Version 4.3.2 (2023.09.1 + 494). Descriptive statistics were used to summarise allele frequencies and sociodemographic characteristics. A comparison of allele frequencies to published data from other African populations was done using the chi-square (χ^2^) test. Logistic regression analysis was used to evaluate the effects of gender, age, ethnicity and HIV status on *NAT2* phenotype. The genotypic distributions at each SNP were assessed for Hardy-Weinberg equilibrium (HWE) using an exact test described by Fung T and Keenan K (eq. 9) [32] and the χ^2^ test. Linkage disequilibrium (LD) was measured using the squared correlation coefficient (*r*^2^) and Lewontin’s standardised disequilibrium coefficient (D′), an indicator of allelic segregation for two genetic variants. The probability level of 0.05 was considered the cut-off value for significance.

## Results

### Sociodemographic characteristics of participants

The sociodemographic characteristics of all the 79 participants included in the study are summarised in Table 1. Their median age (interquartile range) was 39 (12.5) years, with the majority (47%) being ≥40 years. Females were 65% (51/79), those living with HIV were 52% (41/79), and the most predominant ethnic group was the eastern Bantu at 63% (50/79).

**Table 1:** Sociodemographic characteristics of participants (*N* = 79)

Variable	Frequency, *n* = 79
**Gender, n (%)**	
Female	51 (65%)
Male	28 (35%)
**Age groups, n (%)**	
<30	16 (20%)
30–39	26 (33%)
≥40	37 (47%)
**Ethnicity, n (%)**	
Eastern Bantu	50 (63%)
Western Bantu	14 (18%)
Nilotic	11 (14%)
Cushite	4 (5%)
**HIV status, n (%)**	
Negative	38 (48%)
Positive	41 (52%)

### NAT2 sequence analysis

The estimated amplification size of the *NAT2* gene (Exon2) was 1150 bp. Seventy-nine (79) participants were analysed for their genotype at the seven common SNPs of the *NAT2* gene. Five polymorphic sites located within the *NAT2* coding exon were identified, namely 282C > T, 341 T > C, 481C > T, 590G > A and 803A > G.

### Allele and haplotype frequencies

The detected *NAT2* alleles with their frequencies are presented in Table 2. The wild-type *NAT2∗4* was the most frequent allele in 37% (29/79) of the participants. In order of predominance, the allele frequencies of known *NAT2* genetic variants were 282C > T 29% (23/79), 341 T > C 18% (14/79), 803A > G 6% (5/79), 590G > A 6% (5/79) and 481C > T 4% (3/79). Including the reference NAT2*4, we inferred eleven haplotypes (Table 3). In comparison to other diverse African populations represented in published literature, [7, 19, 21, 26, 33, 34] the Kenyan population in this study had a significantly lower frequency of the NAT2*6 (590G > A) allele (χ^2^, *p*< 0.05) (Table 4). Similarly, the studied Kenyan population had significantly lower frequencies of the NAT2*5 (341 T > C) allele (compared to other Bantu and Nilotic populations of Kenya [21] and a Senegalese population [7]), the NAT2*11 (481 C > T) allele (compared to varied African populations represented in the 1000 genome project [34]), and the NAT2*12 (803A > G) allele (compared to the Zulu community [33] and African populations represented in the 1000 genome project [34]) (χ^2^, *p* < 0.05) (Table 4**)**. Additionally, the studied population had significantly higher frequencies of the NAT2*13 (282C > T) allele (compared to a Senegalese population [7]) and the wildtype NAT2*4 allele (compared to a Senegalese population [7], the Zulu community [33] and other diverse African populations inclusive of the East African Community [19]) (χ^2^, *p*< 0.05) (Table 4). Together, these data suggest considerable diversity in the distribution of *NAT2* polymorphisms in populations of African ancestry.

**Table 2:** Frequency of NAT2 alleles (*N* = 79)

NAT2 allele	Mutation site	Amino acid change	Reference ID	Acetylation status	Number of participants (N)	Frequency (95% CI)
NAT2*4	–	WILD	–	Rapid	29	0.367 (0.269–0.477)
NAT2*13	282C > T	Tyr94Tyr	rs1041983	Rapid	23	0.291 (0.203–0.399)
NAT2*5	341 T > C	Ile114Thr	rs1801280	Slow	14	0.177 (0.109–0.276)
NAT2*12	803A > G	Arg268Lys	rs1208	Rapid	5	0.063 (0.027–0.140)
NAT2*6	590G > A	Arg197Gln	rs1799930	Slow	5	0.063 (0.027–0.140)
NAT2*11	481C > T	Leu161Leu	rs1799929	Rapid	3	0.038 (0.013–0.106)

**Table 3:** Frequency of NAT2 haplotypes and predicted phenotype (*N* = 79)

Haplotype	No. of participants	Haplotype frequency	Predicted phenotype^a^
NAT2*4	29	37%	Rapid
NAT2*13A	23	29%	Rapid
NAT2*12 A	5	6%	Rapid
NAT2*11A	3	4%	Rapid
NAT2*5 A	4	5%	Slow
NAT2*5 D	4	5%	Slow
NAT2*5 B	3	4%	Slow
NAT2*5 C	3	4%	Slow
NAT2*6 B	2	3%	Slow
NAT2*6 C	2	3%	Slow
NAT2*6 E	1	1%	Slow

^a^The acetylation phenotype was determined depending on the bimodal distribution pattern based on the Human NAT2 Alleles (Haplotypes) nomenclature

**Table 4:** Frequency of NAT2 alleles compared to published data of other African populations

NAT2 allele	Mutation site	Allele frequencies						
		Kenya (this study)	Kenya [21]^a^	Africa [19]^b^	Africa [26]^c^	Senegal [7]^d^	Zulu [33]^e^	1000 Genome Project [34]^f^
NAT2*4	Wildtype	37%	22%	17%^g^	27%	9%^g^	4%^g^	NA
NAT2*13	282C > T	29%	NA	14%	NA	5%^g^	NA	47%
NAT2*12	803A > G	6%	NA	15%	NA	13%	28%^g^	39%^g^
NAT2*11	481C > T	4%	NA	NA	NA	NA	1%	24%^g^
NAT2*5	341 T > C	18%	38%^g^	27%	33%	36%^g^	7%	29%
NAT2*6	590G > A	6%	25%^g^	26%^g^	30%^g^	NA	NA	24%^g^

^a^NAT2 allele frequencies for Kenya’s Bantu and Nilotic populations [21]

^b^NAT2 allele frequencies for diverse African populations from Kenya, Nigeria, Tanzania and Zimbabwe [19]

^c^NAT2 allele frequencies for diverse African populations from Tanzania, South African Venda, and Zimbabwe [26]

^d^NAT2 allele frequencies for the African ancestry of Madenka in Senegal [7]

^e^NAT2 allele frequencies for Zulu-speaking South Africans [33]

^f^NAT2 allele frequencies for the African ancestry of the Caribbean in Barbados, south-west US, Esan in Nigeria, Gambian in Western Division, the Gambia, Luhya in Webuye, Kenya, Mende in Sierra Leone, Yoruba in Ibadan, Nigeria. The allele frequencies from the different regions did not differ significantly (χ^2^, *p* < 0.05) from each other, so the overall frequency was used for this comparative analysis [34]

^g^Significant difference (χ^2^, *p* < 0.05) in comparison with the proportion of *NAT2* alleles among Kenyan volunteers found in this study

*NA* Not Applicable (Not Determined or Specified)

### Acetylation phenotype

We inferred that 60 (76%) and 19 (24%) of the 79 participants had fast and slow acetylation phenotypes, respectively (Tables 2 and 3). The rapid acetylation phenotype was primarily attributed to the wildtype NAT2*4 genotype (37%) and the NAT2*13 variant (29%), while the slow acetylation phenotype was attributed principally to the NAT2*5 variant (18%) (Tables 2 and 3). Acetylation phenotype differed significantly by gender, with a higher proportion of rapid acetylators being female, 72% vs 28% male, and vice versa (*p* = 0.022, odds ratio [OR] 3.48, 95% confidence interval [CI] 1.21 to 10.48) (Table 5). Our findings suggest a high prevalence of the rapid acetylation phenotype, with gender-associated differences.

**Table 5:** Comparison of the Predicted phenotype by sociodemographic characteristics

Variable	Frequency	Predicted phenotype		*p*-value	OR (95% CI)^a^
	(*N* = 79)	Rapid (*N* = 60)	Slow (*N* = 19)		
**Gender (n, %)**					
Female	51 (65%)	43 (72%)	8 (42%)	–	–
Male	28 (35%)	17 (28%)	11 (58%)	0.022	3.48 (1.21–10.48)
**Age group (n, %)**					
< 30	16 (20%)	13 (23%)	3 (16%)	–	–
30–39	26 (33%)	18 (30%)	8 (42%)	0.394	1.93 (0.46–10.10)
≥ 40	37 (47%)	29 (48%)	8 (42%)	0.813	1.20 (0.29–6.12)
**Ethnicity (n, %)**					
Eastern Bantu	50 (63%)	40 (67%)	10 (53%)	–	–
Western Bantu	14 (18%)	8 (13%)	6 (32%)	0.089	3.00 (0.83–10.76)
Nilotic	11 (14%)	8 (13%)	3 (16%)	0.596	1.50 (0.29–6.33)
Cushite	4 (5%)	4 (7%)	0 (0%)	0.993	–
**HIV status (n, %)**					
Negative	38 (48%)	28 (47%)	10 (53%)	–	–
Positive	41 (52%)	32 (53%)	9 (47%)	0.651	0.79 (0.28–2.22)

^a^ *OR* Odds ratio, *CI* Confidence Interval, Logistic regression analysis

### Genotype frequency

We found heterozygote SNPs to be more common than homozygote SNPs (154 versus 48, respectively), depicting a heterozygous excess. The highest frequency of observed heterozygote genotypes was NAT2*5 341 T > C and NAT2*13  282C > T, occurring equally at 42% (33/79), followed by NAT2*6 590G > A at a frequency of 39% (30/77). The lowest frequency of observed heterozygote genotypes was NAT2*12 803A > G and NAT2*11 481C > T, occurring equally at 37% (29/79).

### Hardy-Weinberg equilibrium and linkage disequilibrium

**Fig. 1:**
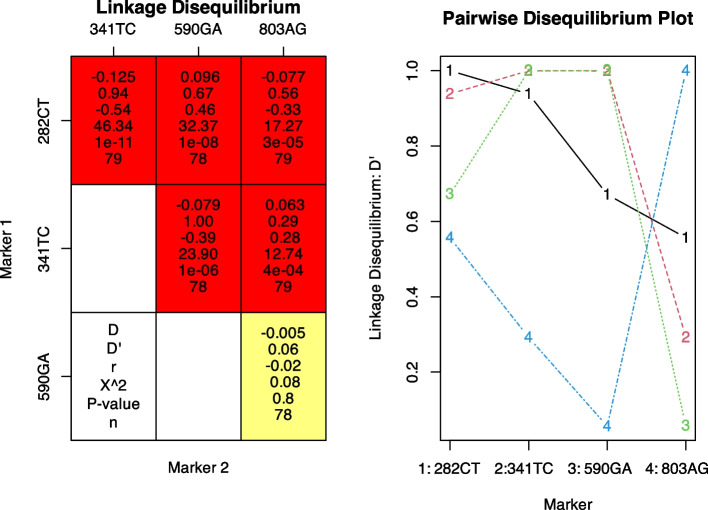
Linkage disequilibrium heatmap (left) and pairwise disequilibrium plot (right). D, Linkage disequilibrium coefficient; D′, Lewontin’s standardised disequilibrium coefficient; r, squared correlation coefficient; X^2, chi-square; n (sample size) = 79, 1 participant had a deletion at mutation site 590G > A. Marker refers to the Single Nucleotide polymorphism (SNPs): 590GA refers to 590 G > A, 341TC refers to 341 T > C, 282CT refers to 282C > T and 803AG refers to 803A > G. Only genetic variants with a frequency higher than 5% were used to evaluate LD. On the left is the LD heatmap; the red squares represent LD, and the yellow square represents no LD. On the right is the pairwise disequilibrium plot. The plots (coloured lines which are numbered) represent the different markers (SNPs), marker 1 being 282C > T, marker 2 being 341 T > C, marker 3 being 590 G > A and marker 4 being 803A > G. The plots that are close together are in moderate to strong LD. For example, 341 T > C (2) and 590 G > A (3) are in strong complete LD and negatively correlated, while 282C > T (1) and 341 T > C (2) are in strong LD and negatively correlated. On the other hand, 803A > G is either in weak LD (with 341 T > C and 282C > T) or no LD (with 590 G > A) as specified

We then examined the HWE for genotypic distribution at each SNP and the LD for the non-random association of alleles at different loci. All five variants were within HWE (based on confidence intervals and *P*-values > 5%; results not shown). Four of the five *NAT2* variants (282C > T, 341 T > C, 590G > A, and 803 A > G) had allele frequencies > 5% and were included in the analysis for LD, as depicted in Fig. 1. *NAT2* 341 T > C variant was in weak negative correlation and strong complete LD with 590G > A (r^2^ = − 0.39, D′ = 1.0, *p*< 0.0001). Similarly, the *NAT2* 341 T > C variant was in weak negative correlation and in strong but not complete LD with the 282C > T variant (r^2^ = − 0.54, D′ = 0.94, *p* < 0.0001). *NAT2* 282C > T was in moderate to weak LD with 590 G > A (D′ = 0.67, r^2^ = 0.46, *p* < 0.0001), and 803A > G (D′ = 0.56, r^2^ = − 0.33, *p* < 0.0001). NAT 2803A > G displayed weak LD and positive correlation with 341 T > C (D′ = 0.29, r^2^ = 0.28, *P*< 0.001) and no LD with 590 G > A (Fig. 1). In summary, although significant pairwise LD was found between the 4 SNPs, the 803A > G displayed weak or no LD with the other SNPs, and 341 T > C was in strong LD with both 282C > T and 590G > A variants. These results suggest constant genetic variation (HWE) and the non-random association of *NAT2* 341 T > C with the 590G > A and 282C > T variants (strong LD).

## Discussion

Genetic variations in the *NAT2* gene affect drug metabolism and modulate therapeutic effectiveness and toxicity. This is particularly important in high TB burden contexts where INH is widely used to treat TB infection (preventive therapy) and disease. INH is partly metabolized through acetylation by the *NAT2* enzyme. Pharmacogenomics testing can potentially improve treatment outcomes with the progressive adoption of genetic analysis to individualize drug therapy [9, 10]. We sequenced the coding region of the *NAT2* gene in 79 participants to identify prevalent SNPs and infer their potential effects on enzyme function. The heterogeneity of this population and the possibility of recombination among the SNPs were also evaluated. Multilocus sequence analysis is essential to deriving accurate genotype information in heterogenous populations such as those from Africa [19, 21, 24]. Therefore, we used the established panel of seven SNPs (rs1801279 (191G > A), rs1041983 (282C > T), rs1801280 (341 T > C), rs1799929 (481C > T), rs1799930 (590G > A), rs1208 (803A > G) and rs1799931 (857G > A)) to infer the NAT2 genotype and phenotype [35, 36].

We found the most frequent haplotype to be the wildtype NAT2^*^4 (37%), and five polymorphic sites were identified within the *NAT2* coding exon. Together with their allelic groups, these include the 282C > T (NAT2^*^13) (29%), 341 T > C (NAT2^*^5) (18%), 803A > G (NAT2*12) (6%), 590G > A (NAT2*6) (6%) and 481C > T (NAT2*11) (4%). These haplotypes and other novel variants have previously been identified in varying proportions among diverse African populations [7, 19, 21, 26, 33, 34]. In keeping with our findings, the NAT2^*^13 haplotype was more predominant than the NAT2*5 and NAT2*6 haplotypes in African populations evaluated in the 1000 genome project. Contrary to our findings, the NAT2*5 and NAT2*6 haplotypes were more predominant overall than the rapid encoding wild-type NAT2*4 and NAT2^*^13 haplotypes in previous studies of varied African populations [7, 19, 21, 26, 33]. From two tribal groups of Kenya, the NAT2*12 (803A > G) variant (one of the least frequent in our study) was the most frequently identified [19]. This was followed by NAT2*11 (481C > T) in one of the tribal groups (the least frequent in our study) [19]. It has long been known that Sub-Saharan Africa has a high sequence variation and haplotype diversity at the *NAT2* gene [7, 19, 21, 24]. Together, these data and prior studies confirm this diversity, thus underscoring the need for more population genetic studies to identify markers of therapeutic variability.

We also inferred the identified polymorphic sites’ potential effects on enzyme function. The rapid acetylation phenotype was prevalent at 76%, largely accounted for by the wild-type NAT2*4 (39%) and the variant NAT2*13A (29%). This contradicts previous findings from Sub-Saharan African populations where the slow acetylation phenotype has been reported as most prevalent, [19, 33] and similar to findings from Asian populations [7]. In keeping with findings from our study, a high frequency of the rapid acetylator phenotype has been reported amongst Nigerians, although the methods used differed [37]. Further, in a Senegalese population, an almost equal number of rapid and slow acetylation phenotypes was reported [22]. Similar to what has been found in many African populations, our study also identified the rapid acetylation haplotype NAT2*12A, which is rarely found in Asian populations [7]. Additionally, the NAT2*5 was the commonest contributor to the slow acetylator phenotype in our study, as is common in many African populations [19, 33]. Acetylation status has been suggested to vary by human adaptation to different exposures, such as dietary habits and varying environments, with agriculturalists depicting slow acetylation phenotype in contrast to hunter-gatherer communities [24, 38, 39]. Our study population was cosmopolitan, consisting of urban immigrants from different geographical settings in Kenya, which may explain the variability from previous reports. Previous data have reported no interaction between gender and *NAT2* Acetylator phenotypes, [40, 41] although the populations studied were not of African ancestry. The small sample size that was predominantly female could be a source of bias in our study. Our findings of a high frequency of the rapid-encoding *NAT2* haplotypes that differ from previous findings may translate to an altered therapeutic efficacy of INH during TB treatment, risking the development of drug resistance. Additionally, there may be gender interactions that need further exploration in context and with larger sample sizes designed to explore associations.

We observed HWE in all five variants. Without evolutionary influences, allele and genotype frequencies in a population will remain constant across generations, according to the Hardy-Weinberg principle [42]. Thus, HWE helps estimate the number of homozygotes and heterozygote variant carriers in genetically stable populations [43]. Deviation from HWE could signify natural selection, excessive inbreeding, mutation, genetic drift, or gene flow [44–46]. Deviation from HWE could also signify genotyping errors in cases of heterozygous excess, more so where no gene reference exists [43, 47]. In our study, sequencing reads were mapped and aligned to the *Homo sapiens* *NAT2* reference, [28] reducing the likelihood of genotyping errors. Our results depict constant genetic variation in the studied population.

We observed strong and complete LD between 341 T > C and 590G > A *NAT2* variants and a strong but incomplete LD between 341 T > C and 282C > T *NAT2* variants. LD evaluates deviation from the random association of alleles [48] and is a key concept to designing ‘indirect’ association studies for complex diseases and identifying genes that may contribute to phenotypic variation [45]. The 341 T > C *NAT2* variant is the main representative of the slow encoding NAT2*5 allele, with the 590G > A variant also indicated as a representative [29]. Our findings of strong and complete LD between 341 T > C and 590G > A suggest that detection of the 590G > A variant may also give an accurate estimation of the slow encoding NAT2*5 genotype in the studied population. On the other hand, the rapid encoding NAT2*13 allele is mainly represented by the presence of the 282C > T variant [29]. None of the other variants identified in our study are indicated as representatives of the NAT2*13 allele [29]. Although we found the 341 T > C variant to be in strong but not complete LD with the 282C > T variant, none of the volunteers with the 282C > T variant also had the homozygous 341 T > C variant. In that regard, we suggest that detection of the 282C > T variant may accurately estimate the rapid encoding NAT2*13 allele in this population. However, despite the high LD observed between the specified SNPs, none could be termed tag SNP (confirmed to be representative) as none had pairwise correlation ≥0.80.

Our study has several limitations. First, the sample size was small, which may create deviation from equilibrium and lead to biased results in association. However, the statistical power indicated that the sample size was sufficient to study the *NAT2* variants in this population. Nevertheless, a larger sample size may be more suited to explore associations. Second, the acetylation phenotype was deduced from the genotype without correlating with phenotypic studies. The ongoing study plans to evaluate this aspect. Despite these limitations, several strengths exist. We used eq. 9, described by Fung T and Keenan K, to account for sampling uncertainty in assessing HWE [32]. We employed all seven signature SNPs within the *NAT2* gene for genotyping. This panel is recognised for accurately typing an individual’s acetylator status. We included both HIV-negative and HIV-positive volunteers in this study. This is important for the generalisability of our study findings, given the wide use of INH in HIV populations for TB treatment and preventive therapy.

## Conclusion

Using multilocus analysis, we identified five polymorphic sites within the *NAT2* gene among Kenyan volunteers with TB infection. None of the SNPs could be termed tag SNP. We report a high frequency of rapid phenotype encoding *NAT2* haplotypes, with gender-associated differences in acetylation phenotype. With these findings, we provide additional genetic characterisation of African populations at *NAT2* that may be useful for developing relevant pharmacogenomic tools for TB therapy. We recommend continued genotyping of African populations at this locus and genotype-phenotype studies, including exploration of gender-associated differences in acetylation phenotypes towards optimised pharmacogenomics-guided TB therapy.

## Data Availability

Datasets supporting the conclusions of this article are available in the GenBank repository, with accession numbers OR138137 to OR138215, https://www.ncbi.nlm.nih.gov/nuccore/OR138137. Sociodemographic data are not publicly available due to privacy policy regulations but are available from the corresponding author upon reasonable request.

## Abbreviations

CI: Confidence interval
HIV: Human immunodeficiency virus
HWE: Hardy-Weinberg equilibrium
INH: Isoniazid
LD: Linkage Disequilibrium
NAT2: N-Acetyltransferase-2
PCR: Polymerase chain reaction
SD: Standard deviation
SNP: Single nucleotide polymorphisms
TB: Tuberculosis.
